# Phase 2 randomized placebo controlled double blind study to assess the efficacy and safety of tecfidera in patients with amyotrophic lateral sclerosis (TEALS Study): Study protocol clinical trial (SPIRIT Compliant)

**DOI:** 10.1097/MD.0000000000018904

**Published:** 2020-02-07

**Authors:** Steve Vucic, Julie Ryder, Linda Mekhael, Henderson RD, Susan Mathers, Merilee Needham, Schultz DW, Kiernan MC

**Affiliations:** a Department of neurology, Westmead Hospital; b Westmead Clinical School University of Sydney, Sydney; c Department of Neurology, Royal Brisbane and Women's Hospital, Brisbane; d Department of Neurology, Calvary Health Care Bethlehem, Melbourne; e Fiona Stanley Hospital, IIID Murdoch University, Notre Dame University and Perron Institute for Neurological and Neurosciences Translational Research; f Department of Neurology, Flinders Medical Centre, Adelaide; g Brain and Mind Center, University of Sydney, Sydney, Australia.

**Keywords:** ALS, ASLFRS-R, neuroimmunity, TECFIDERA, Treg

## Abstract

**Background:**

Amyotrophic lateral sclerosis (ALS) is a progressive and fatal neurodegenerative disorder of the human motor system. Neuroinflammation appears to be an important modulator of disease progression in ALS. Specifically, reduction of regulatory T cell (Treg) levels, along with an increase in pro-inflammatory effector T cells, macrophage activation and upregulation of co-stimulatory pathways have all been associated with a rapid disease course in ALS. Autologous infusion of expanded Tregs into sporadic ALS patients, resulted in greater suppressive function, slowing of disease progression and stabilization of respiratory function. Tecfidera (dimethyl fumarate) increases the ratio of anti-inflammatory (Treg) to proinflammatory T-cells in patients with relapsing remitting multiple sclerosis and rebalances the regulatory: inflammatory axis towards a neuroprotective phenotype. Consequently, the aim of this study was to assess the efficacy, safety, and tolerability of Tecfidera in sporadic ALS.

**Methods:**

The study is an investigator led Phase 2 multi-center, randomized, placebo controlled, double blind clinical trial assessing the efficacy and safety of Tecfidera in patients with sporadic ALS. The study duration is 40 weeks, with a 36-week study period and end of study visit occurring at 40 weeks or at early termination/withdrawal from study. The TEALS study has been registered with the Australian and New Zealand Clinical Trials registry (ANZCTR) under the trials registration number ACTRN12618000534280 and has been approved by the Human Research Ethics Committee and Research Governance Office at the lead site (Westmead Hospital) with the ethics number HREC/17/WMEAD/353. The participating sites have obtained site specific ethics and governance approvals from the local institution.

**Results:**

The primary endpoint is slowing of disease progression as reflected by the differences in the ALS Functional Rating Score-Revised (ALSFRS-R) score at Week 36. The secondary endpoints will include effects in survival, lower motor neuron function, respiratory function, quality of life and safety.

**Conclusion:**

This Phase 2 multi-center, randomized, placebo controlled, double blind clinical trial will provide evidence of efficacy and safety of Tecfidera in sporadic ALS.

## 1 Introduction

Amyotrophic lateral sclerosis (ALS) is a progressive and fatal neurodegenerative disorder of the human motor system, with median survival of 3 to 5 years.^[1,2]^ The currently licensed treatments for ALS include riluzole [anti-glutamatergic agent]^[3–5]^ and edaravone,^[6]^ both of which provide limited benefits. The etiology of sporadic ALS remains unresolved, although a complex multistep interaction between genetic and molecular processes, along with environmental influences, seems likely.^[2,7,8]^ Over the past several decades there have been more than 250 separate clinical studies searching for an effective treatment for ALS, none of which were successful in slowing the progression of ALS.^[9,10]^

There is accumulating evidence for a role of neuroinflammation in ALS pathogenesis, with astrocytes, microglia and T cells actively contributing to neurodegeneration and disease progression in ALS.^[11–16]^ Specifically, reduced expression of mutant superoxide dismutase one (SOD1) protein in microglia^[17]^ and astrocytes^[18]^ has been associated with a slower rate of disease progression. In addition, elimination of functional CD4^+^ T cells accelerates the rate of disease progression in SOD1 mouse models, a process that can be rectified by reconstitution of the immune system through bone marrow transplantation.^[12,19]^

Importantly, upregulation of regulatory T cells (CD4^+^FoxP3^+^ Tregs) in blood and lymph node tissue was reported in the early disease stages of the SOD1 mouse model, with increased expression of FoxP3 mRNA, a key transcriptional regulator of Tregs, evident in the spinal cord.^[20]^ In addition, upregulation of anti-inflammatory cytokines (interleukin [IL]-4, IL-10) is also evident in the early stages of disease resulting in a neuroprotective microglia (M2) phenotype which provides neurotrophic support.^[13]^ With disease progression, there is upregulation of proinflammatory cytokines IL-1β, IL-6 and interferon-γ [IFN-γ]) along with expansion of the CD4^+^CD25^−^ T effector levels, decline in Treg and transition of the microglia to a neurotoxic M1 phenotype.^[13]^ Interestingly, adoptive transfer of Tregs into SOD1 mouse models induces the M2 phenotype and prolongs survival,^[13,21]^ thereby suggesting that Tregs may be neuroprotective in ALS by modulating microglial activation. Of further relevance, expansions of the effector population in the SOD-1 mouse model by IL2 treatment was associated with reduced disease progression, prolonged survival and preservation of motor neuron size and dendritic networks,^[22]^ underscoring a potential therapeutic effect of an immune modulating approach.

Neuroinflammation also appears to be a key modulator of disease progression in ALS patients.^[15,22]^ A reduction of Treg levels, along with an increase in pro-inflammatory effector T cells, increased macrophage activation and upregulation of co-stimulatory pathways have been associated with a rapid disease course in ALS patients.^[23–28]^ Of relevance, rapidly progressive ALS patients express a reduced level of FoxP3 mRNA in the spinal cord along with an increase in peripheral monocytes expressing pro-inflammatory genes.^[15,29]^ Taken together, these findings suggest that the immune balance is skewed to a more proinflammatory state in ALS patients with a rapidly progressive disease course. Importantly, autologous infusion of expanded Tregs into sporadic ALS patients, resulted in greater suppressive function, slowing of disease progression and stabilization of respiratory function,^[30]^ underscoring the therapeutic potential of the Treg based neuromodulatory approach in ALS.

Enhancement of Treg levels in humans can be achieved safely and effectively by using dimethyl fumarate (tecfidera), a commercially available medication for treatment of the relapsing-remitting form of multiple sclerosis.^[31,32]^ In relapsing remitting multiple sclerosis patients, tecfidera was shown to increase the ratio of anti-inflammatory to proinflammatory T-cell subsets.^[33,34]^ Tecfidera also results in stabilization of Treg frequency and a concomitant reduction in pro-inflammatory T-cells [CD4+ INFγ+; CD8+ INFγ+],^[33]^ underscoring its potency in rebalancing the regulatory: inflammatory axis towards a neuroprotective phenotype. Consequently, the aim of the current Phase II randomized placebo controlled multicenter trial was to assess the efficacy and safety of tecfidera in sporadic ALS. Current manuscript publishes the trial protocol (version 3.0) which has been approved by the ethics committee.

## 2 Methods

### 2.1 Aims and objectives

The primary aim of this clinical trial as to assess the efficacy of tecfidera (dimethyl fumarate, DMF) on disease progression in sporadic ALS patients. The secondary aim of this study is to assess effects of tecfidera in sporadic ALS patients on survival, lower motor neuron dysfunction, respiratory dysfunction, quality of life as well as safety and tolerability. The primary and secondary aims of this will be achieved through a multi-center, randomized, double-blind, placebo controlled clinical trial conducted on sporadic ALS patients that will be prospectively recruited from 6 ALS centers in Australia. The total study duration will be 40 weeks, with a 36-week study period and an end of study visit occurring at 40 weeks or at early termination/withdrawal from study.

### 2.2 Primary outcome measure

The primary outcome measure is slowing of disease progression as reflected by the differences in the ALS Functional Rating Score-Revised (ALSFRS-R) score at Week 36 between active and placebo groups. The ALSFRS-R is a validated rating instrument for monitoring the progression of disability in patients with ALS and is utilized for monitoring functional change in ALS patients. The score assesses various 4 domains including:

(i) bulbar function (speech, salivation, swallowing);
(ii) fine motor task (handwriting, cutting food and handling utensils, with or without gastrostomy, dressing and hygiene);
(iii) gross motor task (turning in bed, walking, climbing stairs);
(iv) respiratory function (dyspnea, orthopnea and respiratory insufficiency).^[35]^

Each item within a domain is attributed a score of 0 (complete loss of function) to 4 (normal), yielding a maximum score of 48 when the function is preserved. The ALSFRS-R will be measured at screening, baseline, week 12, 24, 36, and 40.

### 2.3 Secondary outcome measures

The secondary outcome measures will be measured at screening, baseline, week 12, 24, 36 and 40. The secondary endpoints will include measures of efficacy and safety.

1. Survival
  Survival will be defined from symptom onset to death of patients or tracheostomy insertion, whichever occurs first and will be measured in months.
2. Lower motor neuron function
  Lower motor neuron function will be assessed by clinical and neurophysiological biomarkers and will included muscle strength and motor amplitudes.**-Muscle strength**
  (a) *Medical Research Council score*: Clinical assessment of muscle strength will be made by the Medical Research Council (MRC) score. The MRC score is a standard clinical score used in clinical practice and will be performed by the neurologist at each site. This score assesses muscle strength on a 5-point score as follows:
  - 5 = Normal strength.
  - 4 = Active movement against gravity and resistance.
  - 3 = Weak contraction against gravity.
  - 2 = Active movement with gravity eliminated.
  - 1 = Minimal contraction.
  - 0 = Complete paralysis.
  The following movements will be assessed in each patient:
  *-Upper limbs*: Shoulder abduction, elbow flexion, elbow extension, wrist extension, finger extension, finger abduction, and thumb abduction on both sides. The total score when strength normal in the upper limbs will be 70.
  *-Lower limbs*: Hip flexion, abduction, adduction and extension, knee flexion and extension, and ankle dorsiflexion and plantarflexion. The total score when strength is normal in the lower limbs will be 80. The upper and lower limb MRC scores will be combined yielding a maximum score of 150 (0 severe weakness to 150 normal strength).
  (b) *Hand dynamometry*: Muscle strength will also be assessed by hand-held dynamometry (JAMAR PLUS, Patterson Medical, Warrenville, IL). A spring-loaded device that “breaks” at pre-set forces will be used to assess readings obtained by hand-held dynamometry throughout the study. Wherever possible, the same rater will be used at each site for each assessment. Grip strength dynamometry for both hands will be acquired, and the mean force in kilograms will be calculated. Measures will be obtained from each hand in triplicate
  -*Neurophysiological testing:* The degree of lower motor neuron loss will be determined neurophysiological testing performed on nerve conduction study machines (Nicolet EDX, System, Natus, Middleton, WI). The following parameters will be recorded from the intrinsic hand muscles on both sides.
  (a) *Neurophysiological Index (NPI)*: This is an established measure of lower motor neuron dysfunction in ALS.^[36]^ The NPI will be calculated from by stimulating the median nerve at the wrist and recording the motor response from the abductor pollicis brevis muscle using the following formula: 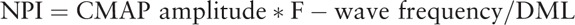  Where CMAP is the compound muscle action potential, DML is distal motor latency and F-wave frequency is the number of F-wave that occur with 10 consecutive suprathreshold stimulations.
  (b) *Split Hand Index (SI)*: The split hand index as an objective biomarker of the split hand phenomenon, an important clinical feature of ALS.^[37–39]^ The SI will be calculated as follows: 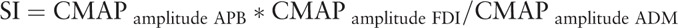
3. Respiratory function
  Respiratory function will be assessed by using specific respiratory function tests.
  -*Forced vital capacity (FVC)*: Forced expiratory volume (FEV) measures how much air a person can exhale during a forced breath. The amount of air exhaled may be measured during the first (FEV1), second (FEV2), or third seconds (FEV3) of the forced breath. Forced vital capacity (FVC) is the total amount of air exhaled during the FEV test. The definition of FVC is therefore, the amount of air which can be forcibly exhaled from the lungs after taking the deepest breath possible. For measuring FVC the patient will be instructed to inhale deeply and exhales completely while in a standing position. Results of the test will be recorded and analyzed by the spirometry machine (Welch-Allyn) and interpreted by the PI. Three trials will be performed and the best FVC result (% of predicted) will be recorded.
  *-Sniff nasal inspiratory pressure (SNIP)*: The SNIP is the measurement of pressure through an occluded nostril during sniffs performed through the contralateral nostril. It is an accurate and non-invasive approximation of oesophageal pressure swing during sniff manoeuvres. Respiratory strength is assessed by measuring the maximal inspiratory pressure (MIP or PI_max_) and the maximal expiratory pressure (MEP or PE_max_). The MIP reflects the strength of the diaphragm as well as other inspiratory and expiratory muscles.
4. Urinary neurotrophin receptor P75 level
  The urinary neurotrophin receptor p75 is a biomarker of neurodegeneration measured by an enzyme-linked immunosorbent assay (ELISA).^[40]^ Specifically, the ELISA test measures the extracellular portion of the neurotrophin receptor p75 that is cleaved from neurons in ALS. Neurotrophin receptor p75 is increased in serum, CSF and urine of ALS patients, although urinary measures are preferable. With disease progression in ALS, there is an increase in neurotrophic p75 levels.^[41,42]^
  Urinary neurotrophic receptor p75 levels will be measured according to the following protocol:
  - The test will be performed on diluted urine and will be performed in triplicate in 4 separate assays. The mean value is calculated from the 12 measurements.
  - Urinary dilution is accounted for by correcting with creatinine and the level of p75 (ng) will be provided per mg of creatinine.
  - A mid-stream urine sample will be collected into a sterile urine container.
  - The urine samples will undergo urinalysis (SIEMENS Multistix 10 SG) and samples with abnormal readings (high glucose, pH > 8, positive for leucocytes or blood) will be discarded. Eligible urine samples 10–50 mL) will be centrifuged at 2000 g (4°C) for 5 minutes. A 1 mL aliquot will be collected, placed in secure tube, labeled and stored at −80°C within 4 hours of collection) until transported to the ALS Biomarker Centre at Flinders University for analysis.
5. ALS Specific Quality of Life Revised (ALSSQOL-R)
  The ALS Quality of Life Revised is a validated questionnaire that will be used to assess the impact of tecfidera on the patients quality of life.^[43]^ The ALSSQOL-R is a 50-item disease-specific questionnaire covering multiple domains. Each question is scored from 0 (not at all) to 10 (very much) yielding a total score range from 0 to 500. The questionnaire can be completed by most ALS patients in approximately 15 to 20 minutes.
6. Assessment of Safety
  The Investigators and study site staff will be responsible for documentation of events meeting the definition of an adverse event (AE) or serious adverse event (SAE). Adverse events will be defined any unfavorable and unintended consequence (including an abnormal laboratory finding), symptom, or disease (new or exacerbated) temporally associated with the use of tecfidera, whether or not considered related to the medicinal product. Disease progression of ALS will not be considered an AE. The potential AEs related to tecfidera are highlighted in Table 1.

**Table 1:**
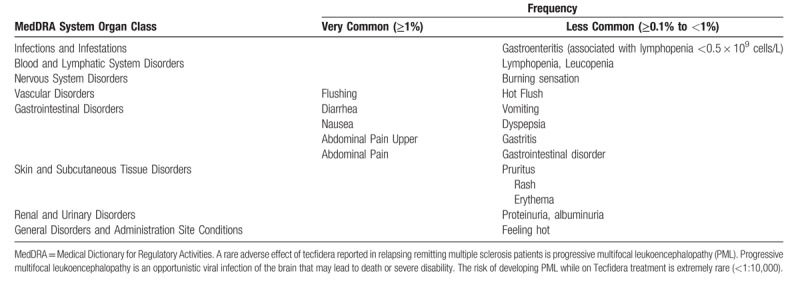
Adverse reactions reported with tecfidera.

Serious adverse events are defined as any event that results in death, disability or incapacity, is life threatening, or leads to congenital abnormality/birth defect. Hospitalization for any medical event, other than elective surgery, will also be deemed an SAE. All opportunistic infections and malignancies will be considered as SAEs. The investigator will make an assessment of the seriousness and causality of each AE and SAE.

### 2.4 Patient enrolment and withdrawal

*Sample size calculation*: Based on previous clinical studies and literature,^[44,45]^ it was assumed that the mean difference in the ASLFRS-R score between active and placebo group at 36 weeks will be 7 [common standard deviation of 8%]. A clinically important response rate on Tecfidera was defined as 0.9, and with a 2:1 (Tecfidera:Placebo) allocation, 90% power, 5% statistical significance rate and 20% dropout rate, we estimated that 90 patients (60:30) need to be randomized inorder to attain the primary outcome measure. The inclusion and exclusion criteria are outlined in Table 2.

**Table 2:**
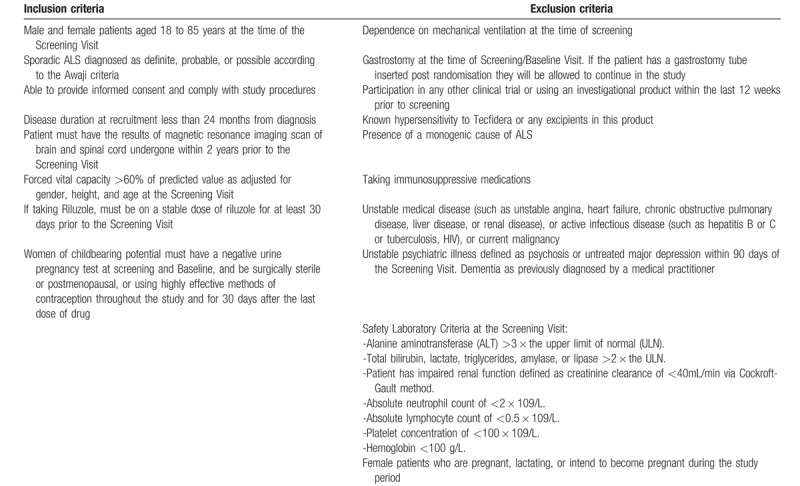
The inclusion and exclusion criteria for TEALS study.

## 3 Study design

Investigator led Phase II multi-center, randomized, placebo controlled, double blind clinical trial assessing the efficacy and safety of Tecfidera in patients with sporadic ALS. Study procedures will be performed as detailed in the Schedule of Events Flow Chart in Table 3.

**Table 3:**
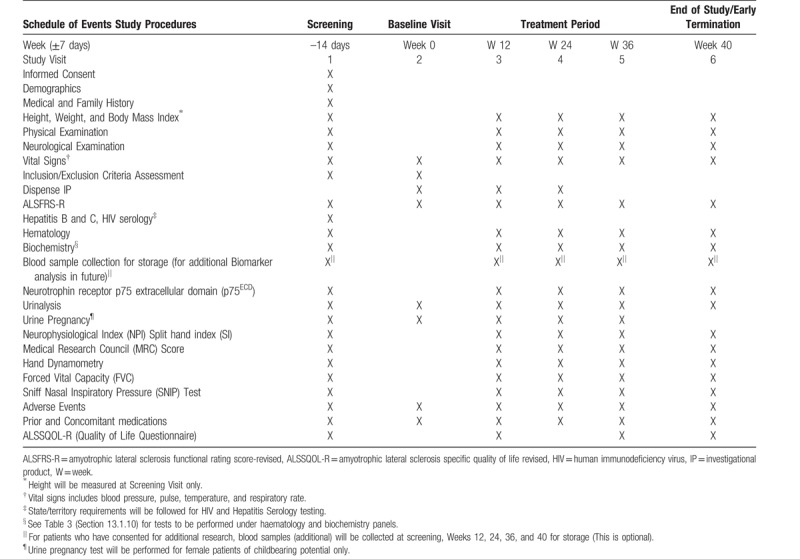
Schedule of events for the TEALS study.

### 3.1 Screening Visit (Visit 1) -14 days

Patients will be screened for the study after signing an approved Informed Consent Form (ICF). As part of the screening, patient's extensive medical and neurological history will be recorded. Inclusion/exclusion criteria will be evaluated and the vital signs (blood pressure, pulse, temperature, and respiratory rate) will be recorded. Physical and neurological examinations will be performed. Patient's height and weight will be measured and body mass index (BMI) will be calculated. The visit will also involve an initial assessment of ALSFRS-R, NPI and SI, FVC as measured by handheld spirometer, SNIP test, quantitative hand muscle testing by dynamometry, and global strength using MRC score. Prior/ongoing medications will be recorded. All patients will undergo laboratory evaluation of hematological and biochemical parameters; hepatitis B, C, and human immunodeficiency virus (HIV) serology; and urinalysis. In addition, women of childbearing potential will undergo a urine pregnancy test. Urine will also be collected for neurotrophin receptor P75 levels (secondary outcome). An ALSSQOL-R questionnaire will be completed by the patient at screening.

### 3.2 Baseline (Visit 2) Week 0 (14 days post visit 1)

All patients will have their inclusion and exclusion criteria checked at the Baseline Visit (Week 0) and eligible patients will be randomized to either active group (Tecfidera) or control group (placebo) in a 2:1 ratio and the IP will be dispensed. Randomization will be performed by a third party (Cenduit) working with the CRO, using an Interactive Web Response System with random number generation. The numbers will be emailed to a designated pharmacist at each sit and bottles will be labeled by the randomly generated number. All parties at each site will be blinded, and unblinding will only occur after data analysis. This study visit will also involve an assessment of ALSFRS-R, vital signs (blood pressure, pulse rate, temperature, and respiratory rate), and any changes to concomitant medications. Urine samples will be collected for safety monitoring (urinalysis). In addition, women of childbearing potential will undergo another urine pregnancy test.

### 3.3 Treatment Period (Visits 3, 4, and 5) Weeks 12, 24, and 36

Patients will return to the site at Weeks 12, 24, and 36 for neurological and physical examinations. Their weight, BMI, and vital signs (blood pressure, pulse, temperature, and respiratory rate) will be recorded. The visit will also involve an assessment of ALSFRS-R, NPI and SI, FVC as measured by handheld spirometer, SNIP test, quantitative hand muscle testing by dynamometry, and global strength as measured by the MRC score. All patients will undergo laboratory evaluation of hematological and biochemical parameters as well as urinalysis. In addition, women of childbearing potential will undergo a urine pregnancy test and urine will also be collected to test neurotrophin receptor P75 levels (secondary endpoint). If the patient consented for additional research in future, blood samples (additional) will be collected for storage. At Week 12 and 36, an ALSSQOL-R questionnaire will be completed by patients and the IP will be dispensed at Week 12 and Week 24.

### 3.4 End of Study (Visit 6) Week 40

After completing Week 36 procedures, all patients will return to the study site at Week 40 for the End of Study Visit (4 weeks after the end of treatment visit). Patients who discontinue the study early will be asked to return to the study site for the early termination procedures 4 weeks after their last dose of Tecfidera/placebo. At this visit, neurological and physical examination will be performed and vital signs (blood pressure, pulse, temperature, and respiratory rate) will be recorded. The visit will also involve an assessment of ALSFRS-R, NPI and SI, FVC as measured by handheld spirometer, SNIP test, quantitative hand muscle testing by dynamometry, and global strength using MRC score. All patients will undergo laboratory evaluation of hematological and biochemical parameters as well as urinalysis. Urine will also be collected to test neurotrophin receptor P75 levels (secondary endpoint). An ALSSQOL-R questionnaire will also be completed by all patients. Adverse events and changes to concomitant medications will be observed and evaluated.

### 3.5 Safety monitoring

An independent data safety management board (DSMB) will oversee the safety aspects of this study. The DSMB will also include independent specialist physicians from different medical subspecialties. The DSMB will periodically examine the safety data emerging from the study and provide its recommendations to the chief investigator (CI) who will then pass these onto the independent ethics committee (IEC). The roles and responsibilities of the DSMB, their operational procedures, and method of communication with the CI will be described in a separate DSMB charter. Members of DSMB will not be investigators in the study nor will they have any conflict of interest with the CI.

The clinical trial may be prematurely suspended or terminated if, in the opinion of the DSMB or PIs, there is reasonable cause. Written notification, documenting the reason for study suspension or termination, will be provided by the suspending or terminating party. The pertinent regulatory authorities will be informed according to the national regulations.

Circumstances that may warrant termination include:

- Determination of unexpected, significant, or unacceptable risk to patients.
- Insufficient adherence to protocol requirements.
- Data that are not sufficiently complete and/or evaluable.
- Plans to modify, suspend, or discontinue the development of the IP.

If the study is prematurely terminated or suspended, the CI will promptly inform the site-specific PIs, their institutions, and the regulatory authority of the termination or suspension and the reason(s) for the termination or suspension. The IEC will also be informed promptly and provided the reason(s) for the termination or suspension by the Sponsor or by the CI/institution, as specified by the applicable regulatory requirement(s).

Patients will be free to withdraw from the study at any time for any reason without penalty to their continuing medical care. In addition, patients must be withdrawn from the study by the site PI in consultation with the CI for the following reasons:

- Grade 4 clinical adverse events considered causally related to Tecfidera.
- Allergic or anaphylactic reactions to IP.
- Development of severe lymphopenia (persistent lymphocyte count <0.5×10^9^ cells/L, for 8 weeks).
- Pregnancy.
- Compliance failure.
- Protocol violation.
- Serum creatinine >1.2 × upper limit of normal (ULN).
- Alanine aminotransferase (ALT) >5 × ULN.
- Single aspartate aminotransferase (AST) >8 × ULN.
- The ALT or AST levels >3 × ULN for 4 consecutive weeks.
- ALT or AST >3 × ULN and (total bilirubin >2 × ULN).
- ALT or AST >3 × ULN with appearance of fatigue, nausea, vomiting, right upper quadrant pain or tenderness, fever, rash, and/or eosinophilia (>5%).

Every effort will be made to ensure that patients who do not complete all study requirements return to the site for the Early Termination Visit, 4 weeks after the last dose of IP. Patients who withdraw due to an AE or SAE will be given appropriate care under medical supervision until the symptoms resolve or the patient's condition becomes stable.

### 3.6 Statistical methods

The statistical analysis will be performed using statistical analysis system (SAS) Version 9.4. All details regarding the statistical analysis and the preparation of tables, listings, and figures will be described in the Statistical Analysis Plan (SAP) and approved by the Sponsor before database lock. The default summary statistics for continuous variables include number of contributing observations (n), mean, SD, median, minimum, and maximum. For categorical variables, the number and percentage (in each category will be the default summary presentation. “Baseline” is defined as the last observed value of the parameter of interest prior to the first intake of IP (this includes unscheduled visits). For numerical variables, change from Baseline will be calculated as the difference between the value of interest and the corresponding Baseline value. All formal statistical tests will be two-sided at the 5% significance level. Point estimates of treatment differences will be accompanied with a two-sided 95% confidence intervals. In the case of normality assumption violations, appropriate non-parametric methods will be used for analysis. All data will be presented in by-patient listings.

The following analysis populations will be defined as follows:

i. *Intent-to-Treat (ITT) Population*- The ITT population will include all randomized patients. In this population, treatment will be assigned based upon the treatment arm to which patients were randomized regardless of which treatment they received;
ii. *Safety Population (SAF)*- The SAF includes patients who are randomized and took at least 1 dose of the IP. The safety analysis will be conducted according to the treatment that a patient actually receives;
iii. *Per Protocol Population (PP)-* The PP population will include all patients who do not have major protocol deviations that would affect the evaluation of the primary efficacy endpoint.

The primary endpoint (ALSFRS-R change at 36 weeks) will be analyzed using Mixed Model for Repeated Measures with treatment as fixed effect and Baseline ALSFRFS-R score as covariate. For the active treatment versus placebo comparison, the results will be presented in terms of an estimated treatment difference together with its associated 95% confidence intervals and 2-sided *P* value. Possible effects of other covariates will also be investigated. Missing data handling and possible sensitivity analysis will be described in the SAP. Details of specific alternative statistical methods in the event model assumptions are violated will be documented in the SAP.

Efficacy analysis for secondary endpoints (see outcome measures section) will be summarized and evaluated using the ITT population. Safety analyses will be performed using SAF. All safety data will be provided in by-patient listings. The safety variables that will be analyzed are outlined in Table 1. No interim analysis is planned for the study. All statistical analysis has been performed by the IQUVIA (CRO) biostatisticians in consultation with the PIs (Vucic and Kiernan).

## 4 Ethics and dissemination

The TEALS study has been approved by the Human Research Ethics Committee and Research Governance Office at the lead site (Westmead Hospital) with the ethics number HREC/17/WMEAD/353. The participating sites have obtained site specific ethics and governance approvals from the local institution. Any protocol modifications will be communicated by the PI (SV) to lead sites via email after ethics approval.

Patients, legally authorized representatives, caregivers or responsible family members, may provide written consent on the approved ICF. The PI or research staff designee will explain all study procedures, risks, and alternative therapies to the patient. The patient will have an opportunity to have all questions answered. The appropriate parties will then sign and date the ICF, indicating willingness to participate in the study. A copy of the signed ICF will be given to the patient.

All data will be stored in a purpose-built data base for the TEALS study. The data base was developed by the contract research organization (IQUVIA) in consultation with the chief and principle investigators. Upon completion of the study (including data lock and analysis), unblinded data will be stored for a minimum of 15 years on the database to which the CI and PI will have access. The results will be disseminated by presentations at neurology scientific meetings and through manuscript publications in international neurology or medical journals. The results will be published irrespective of whether the primary and secondary endpoints are attained. All PIs will be authors on the trial. The data set and statistics will be made available upon reasonable request.

## 5 Trial registration

This study has been registered with the Australian and New Zealand Clinical Trials registry (ANZCTR) under registration number ACTRN12618000534280.

## 6 Trial status

The TEALS study commenced in October 2017 and is ongoing. We anticipate that the results will be available in the last quarter of 2020 or first quarter of 2021.

## 7 Discussion

There is increasing evidence for an important role of neuroinflammation in regulating disease activity in ALS.^[46]^ Regulatory T cells appear to be important in regulating the rate of disease progression in ALS,^[13]^ as evidenced by significant correlation between the rates of disease progression and Treg levels,^[13]^ as well as neuroprotective effects exerted by Tregs in mouse models of ALS.^[28]^ In addition, reconstitution studies in mutant SOD1 muse models have also demonstrated that CD4^+^ T cells improve neurological function and prolong survival, with the events being most evident when Tregs were passively transferred, thereby implying an important neuroprotective role for the Treg subsets.^[12,13,19,21]^

In ALS patients, memory effector Tregs (CD45RO^+^) levels exhibit significant correlations with the rates of disease progression.^[22]^ These findings suggest that the functional capacity of Tregs to induce immunosuppression is critical for reducing the rates of disease progression in ALS. Importantly, autologous infusion of expanded Tregs (which exhibit normal suppressive function) into ALS patients resulted in a slowing of disease progression and stabilization of respiratory function.^[30]^ Given that Tecfidera can enhance Treg levels in patients with relapsing remitting multiple sclerosis,^[33,34]^ the aim of the current trial was to assess the efficacy and safety of tecfidera in sporadic ALS.

## Author contributions

**Conceptualization:** Steve Vucic, Matthew Kiernan.

**Data curation:** Steve Vucic, Julie Ryder, Linda Mekhael, Matthew Kiernan.

**Formal analysis:** Steve Vucic, Matthew Kiernan.

**Funding acquisition:** Steve Vucic, Matthew Kiernan.

**Investigation:** Steve Vucic, Robert Henderson, Susan Mathers, Merilee Needham, David Schultz, Matthew Kiernan.

**Methodology:** Steve Vucic, Robert Henderson, Susan Mathers, Merilee Needham, David Schultz, Matthew Kiernan.

**Project administration:** Steve Vucic, Robert Henderson, Susan Mathers, Merilee Needham, David Schultz, Matthew Kiernan.

**Resources:** Steve Vucic, Matthew Kiernan.

**Software:** Steve Vucic, Julie Ryder, Linda Mekhael, Matthew Kiernan.

**Supervision:** Steve Vucic, Julie Ryder, Linda Mekhael, Matthew Kiernan.

**Validation:** Steve Vucic, Matthew Kiernan.

**Visualization:** Steve Vucic, Julie Ryder, Linda Mekhael, Matthew Kiernan.

**Writing – original draft:** Steve Vucic, Julie Ryder, Linda Mekhael, Matthew Kiernan.

**Writing – review & editing:** Steve Vucic, Robert Henderson, Susan Mathers, Merilee Needham, David Schultz, Matthew Kiernan.
